# Recent advancements in double-expressor lymphoma: novel therapeutic approaches and prospects

**DOI:** 10.1093/oncolo/oyaf085

**Published:** 2025-06-17

**Authors:** Yuejian Zhuo, Dongdong Zhang

**Affiliations:** Department of Oncology, Xiangyang No. 1 People’s Hospital, Hubei University of Medicine, Xiangyang 441000, China; Department of Oncology, Xiangyang No. 1 People’s Hospital, Hubei University of Medicine, Xiangyang 441000, China

**Keywords:** double-expressor lymphoma, activated B-cell-like, international prognostic index, diffuse large B.cell lymphoma

## Abstract

Double-expressor lymphoma (DEL) is a newly identified special subtype of diffuse large B-cell lymphoma (DLBCL), which is predominantly found in the activated B-cell-like (ABC) subtype of DLBCL. Characterized by concurrent overexpression of BCL2 and MYC, DEL is associated with poorer prognosis. Standard chemoimmunotherapy can achieve clinical cure in nearly 70% of DLBCL cases. DEL mainly presents with intermediate-to-high-risk international prognostic index scores, advanced stage at diagnosis, and may involve specific chromosomal rearrangements, mainly influencing older patients. These factors are interconnected and contribute to less favorable treatment outcomes. We review emerging drugs and clinical trial data potentially effective against DEL, formulating treatment recommendations based on evidence levels to provide a theoretical foundation for the clinical treatment of DEL.

**Implications for Practice**

1. We summarized effective medications for DEL treatment and elucidated their mechanisms of action.
2. We reviewed clinical trials demonstrating efficacy in DEL treatment and established recommendations based on clinical outcomes.

## Introduction

Diffuse large B-cell lymphoma (DLBCL) was originally classified into 2 major categories based on molecular cell of origin (COO): germinal center B cell (GCB) type and activated B cell (ABC) type. The 2016 revision of the World Health Organization (WHO) classification of lymphoid neoplasms proposed a new subtype of DLBCL, double-expressor lymphoma (DEL), which is characterized by dual overexpression of BCL2 and MYC proteins without underlying chromosomal rearrangements. The recommended criteria for defining these cases include a cutoff of 40% MYC-expressing cells and over 50% BCL2 expression, as determined by immunohistochemical analysis.^1^

The estimated incidence of DEL in DLBCL is 31%. It is more frequent in ABC DLBCL, accounting for about 63% of ABC DLBCL cases, compared with only 37% of GCB cases.^2^ Previous studies have confirmed that patients with GCB DLBCL had a superior survival. However, in the GCB subtype, the overall survival (OS) rate in the DEL group was significantly lower than that in the non-DEL group.^3,4^ Additionally, overexpression of MYC and BCL-2 independently represents a high-risk factor for central nervous system relapse, leading to unfavorable OS.^5,6^

Standard chemoimmunotherapy with rituximab, cyclophosphamide, doxorubicin, vincristine, and prednisone (R-CHOP) achieves a cure rate of ~70% in DLBCL.^7^ The curability of DLBCL is significantly affected by various factors, including age, International Prognostic Index (IPI) score, COO subtypes, and the presence/absence of specific chromosomal rearrangements.^8^ However, patients with DEL are more frequently found in the elderly population and are more likely to present with advanced-stage disease, intermediate-to-high-risk IPI scores, and a higher Ki-67 proliferative index.^4,9^ Furthermore, nearly 24% of cases with DEL exhibit concurrent P53 overexpression, exerting a negative prognostic impact on DLBCL patients.^10^ These adverse factors result in a lower complete remission (CR) and cure rate in patients following R-CHOP treatment,^2^ as well as a significant reduction in progression-free survival (PFS) and OS.^11^ Thus, some scholars have attempted to use the intensified R-DA-EPOCH regimen as an alternative to the R-CHOP regimen to improve the clinical outcome. Nevertheless, there is still controversy about whether this regimen can provide survival benefits. Some studies suggest that the DA-EPOCH regimen can extend PFS and improve prognosis for patients aging under 65 years old with DEL and those with DEL with a single rearrangement (*BCL2* or *MYC*).^12,13^ However, the DA-EPOCH regimen does not confer significant benefits to DEL patients with TP53 mutations or those with double-hit or triple-hit scenarios.^12^ Additionally, some researchers demonstrated that the DA-EPOCH regimen does not improve DEL patients’ prognosis.^14^ Conversely, the R-CHOP regimen, coupled with central prophylaxis, exhibited to be a successful and curative treatment for a substantial proportion of DEL patients.^13,15,16^

The majority of DEL patients are elderly (over 60 years old) and typically present with a high Ki-67 index (>80%).^17,18^ Both R-CHOP and DA-EPOCH are intensive chemotherapy regimens, and the frequent or severe chemotherapy-related adverse effects can render some patients to be intolerant. This intolerance may lead to treatment discontinuation or interruptions, resulting in suboptimal therapeutic outcomes. Therefore, the development of new, effective, and less toxic treatment regimens is crucial. This study provided a concise overview of emerging treatments for DEL, aiming to develop optimal management strategies for DEL.

## The Bruton’s Tyrosine Kinase inhibitors (BTKi)

B-cell receptor (BCR)-mediated signaling plays notable role in the generation and maintenance of B lymphocytes and the pathogenesis of DLBCL.^19^ Bruton’s Tyrosine Kinase (BTK) is a key molecule in the BCR signaling pathway. Bruton’s Tyrosine Kinase is phosphorylated and activated by tyrosine kinases (SYK and LYN) following BCR activation. Activated BTK further promotes BCR signal transduction by recruiting and activating downstream PLCγ2, which in turn activates the NF-κB and MAPK signaling pathways, further promoting the expression levels of MYC and BCL2.^20^ B-cell receptor is hyperactivated in DEL, and the degree of activation is positively correlated with the expression levels of MYC and BCL2.^21^ Notably, BTKi can inhibit kinase activity by binding to the cysteine residues in the active site of BTK, thereby inhibiting the BCR signaling pathway. According to the aforementioned molecular mechanisms, BTKi holds promise in the treatment of DEL.

Studies have assessed the therapeutic efficacy of BTKi in DEL patients. A prospective, single-arm, phase 2 clinical study (*n* = 28) on newly diagnosed DE-DLBCL patients indicated that after 6 cycles of ZR-CHOP (Zanubrutinib plus R-CHOP) treatment, the response rate was significantly higher compared with DE-DLBCL patients who were previously treated with R-CHOP (85.7% vs 70%).^22^ Another phase II clinical trial included 8 treatment-naive patients with DEL. After receiving 6-8 cycles of Orelabrutinib combined with the R-CHOP regimen, all patients achieved CR.^23^ A phase III trial revealed that patients with high BCL2/MYC co-expression had inferior survival outcomes than those without high BCL2/MYC co-expression when treated with placebo plus R-CHOP. However, no significant difference was observed in event-free survival (EFS) and OS when treated with ibrutinib-R-CHOP, suggesting that incorporating BTKi might have exhibited potential to improve double-expressor patients’ poor prognosis.^24^ These promising findings indicated that BTKi could become a significant treatment option for DEL patients. However, the combination of BTKi and R-CHOP has led to more frequent or severe treatment-related adverse reactions. In the phase III PHOENIX trial, the ibrutinib plus R-CHOP group showed a higher incidence of serious adverse events compared with the placebo plus R-CHOP group (53.1% vs 34.0%), particularly febrile neutropenia, diarrhea, cytopenia, and pneumonia. Moreover, adverse events leading to treatment discontinuation were more frequent in the ibrutinib plus R-CHOP group (31.5% vs 13.6%), and the long-term prognosis remains uncertain.^25^ Therefore, expanding sample studies and ongoing follow-up are essential to clarify these outcomes.

## Histone deacetylase inhibitors (HDACi)

The potential epigenetic dysregulation driving tumorigenesis and facilitating the development of DLBCL involves histone deacetylases (HDACs).^26^ These enzymes are responsible for the deacetylation of histones, emerging advantageous to maintain chromatin stability. Overactivity of HDACs can lead to the formation of a compacted and transcriptionally repressed chromatin structure, thereby contributing to lymphomagenesis.^27^ HDACs contribute to the process where MYC activation mediates the transcriptional repression of the miR-15 and let-7 families in lymphoma. The inhibition of HDACs can relieve this repression and upregulate miR-15 and let-7, downregulating BCL2 expression level and promoting tumor apoptosis.^28^ Selective inhibition of HDAC3 restores immunosurveillance in CREBBP-mutant DLBCL.^29^ Moreover, HDACi can downregulate the expression levels of MYC, BCL2, and BCL-x genes by inhibiting the HDAC/STAT3/Bcl-2 and JAK2/STAT3 signaling pathways, promoting the apoptosis of cancer cells.^30,31^ Thus, HDACi can be potential therapeutic drugs for DEL.

A phase II clinical study evaluated the efficacy and safety of combining the HDAC inhibitor tucidinostat (Chidamide) with R-CHOP (CR-CHOP) in elderly patients who were diagnosed with DLBCL. The study enrolled 49 patients, including 12 who were diagnosed with DEL. All DEL patients achieved CR, with significantly improved 2-year PFS rate (83% vs 46%) and OS rate (92% vs 63%) compared with historical controls.^32^ Although all elderly patients had an IPI score of ≥2 points, no grade IV adverse reactions occurred during treatment. A recent randomized, double-blind, multicenter, phase III trial (*n* = 423) on the safety and efficacy of CR-CHOP for the treatment of previously untreated DEL patients indicated that the CR rate (73% vs 61.8%) and 2-year EFS rate (58.9% vs 46.2%) were significantly higher in the CR-CHOP group compared with those in the control group, along with a manageable safety profile.^33^ These two studies demonstrated that CR-CHOP regimen could be a feasible and efficacious novel approach for treating DEL. However, further compelling clinical research is required to confirm this outcome, as well as longer follow-up to determine whether it can improve long-term prognosis.

## The BCL-2 inhibitor

BCL-2 is a key regulator of the apoptotic process. Selective inhibition of BCL-2 emerged promising for the treatment of BCL-2-dependent hematological malignancies. As a selective antagonist of BCL-2, Venetoclax relieves the inhibition of Bax and Bak by competitively inhibiting the binding of BCL-2 to BIM, further leading to the apoptosis of tumor cells. Preclinical studies revealed that Venetoclax demonstrated specific ability of killing MYC+/BCL2 + lymphoma cells by enhancing antigen-specific effector T-cell responses and antagonizing BCL-2.^34^ Venetoclax also exhibits synergistic anti-tumor effects when combined with rituximab and HDACi in DEL mice.^35,36^ These noticeable results have attracted clinicians to explore the applicability of BCL-2 inhibitors in the treatment of DEL.

A phase I study explored the efficacy, pharmacokinetics, and safety of Venetoclax in relapsed/refractory (R/R) non-Hodgkin lymphoma (NHL). Among 6 DEL patients with evaluable tumors, 2 achieved an objective response.^37^ The results of another phase I trial revealed that 87.5% (*n* = 7/8) of DEL patients achieved CR after treatment with Venetoclax plus R-/G-CHOP.^38^ A phase II clinical trial evaluated the efficacy and safety of Venetoclax plus R-CHOP in patients with DLBCL compared with historical controls treated with R-CHOP. The study demonstrated that venetoclax combined with R-CHOP improved survival in DLBCL patients with BCL-2 protein overexpression. However, no significant improvement in PFS or OS was observed in DEL patients treated with Venetoclax plus R-CHOP.^39^ The results of these preliminary clinical trials demonstrated that DEL patients may potentially benefit from regimens containing Venetoclax, while large-scale phase III clinical studies are still needed to confirm these findings.

## Proteasome inhibitors and immunomodulatory agents

Proteasome inhibitors (PIs) can repress the degradation of phosphorylated IκB protein, thereby preventing NF-κB from entering the nucleus and activating the transcription of genes that promote cell survival. This suggests that PIs may be effective in the treatment of DEL by potentially inhibiting MYC and BCL2 through suppression of NF-κB activation. Bortezomib is a reversible PI that may enhance the activity of chemoimmunotherapeutic agents in B cell lymphoma. Three representative clinical trials have been conducted to evaluate its efficacy in combination with R-CHOP (BR-CHOP) for previously untreated DLBCL patients, and it confirmed the efficacy of BR-CHOP.^40^ However, data from one phase II and another phase III clinical trials (REMoDL-B) indicated that BR-CHOP did not improve OS and PFS within a follow-up period of 30-34 months.^7,41^ It was found that patients with ABC-type DLBCL in the BR-CHOP group benefited from the improved PFS and OS rates after a 5-year follow-up, and this benefit was also observed in DEL patients.^42^ Bortezomib can provide a long-term survival benefit in ABC-DLBCL and DEL.

Lenalidomide, an immunomodulatory agent, has exhibited notable therapeutic effects on relapsed DLBCL patients. Previous studies have demonstrated that Lenalidomide could downregulate the expression levels of transcription factors IRF4 and SPIB, inhibiting the activation of CARD11, thereby suppressing NF-κB signaling pathway. This suggests that Lenalidomide may exhibit a notable therapeutic efficacy in DEL by targeting NF-κB activity. Clinical trials on the Lenalidomide plus R-CHOP (R2-CHOP) have not yielded consistent results. Two phase II clinical trials demonstrated that the R2-CHOP regimen could be advantageous for elderly patients.^43,44^ However, a large phase III clinical trial (ROBUST) failed to show any benefit.^45^ Maintenance therapy with Lenalidomide could improve PFS in patients who responded to R-CHOP, while OS was not improved.^46^ A recent phase II SMART trial demonstrated that Lenalidomide in combination with BTKi and rituximab achieved an impressive CR rate of 95%, a 2-year PFS rate of 91%, and an OS rate of 97% in newly diagnosed DLBCL, and 62% of patients were diagnosed with DEL.^47^ These findings highlighted the potential therapeutic influences of immunomodulatory agents as part of combination therapy on DEL patients.

## Antibody-drug conjugates

Polatuzumab vedotin (Pola) is a novel antibody-drug conjugates (ADC) consisting of a CD79b-directed antibody linked to monomethyl auristatin E. Pola in combination with R-CHP (Pola-R-CHP) has been approved as a first-line treatment for previously untreated and R/R DLBCL patients. A phase III clinical study (POLARIX) on newly diagnosed DLBCL patients revealed that the Pola-CHP group demonstrated a 12.4% improvement in the 2-year PFS rate compared with the R-CHOP group (75.5% vs 63.1%, hazard ratio [HR] = 0.6) in DEL patients.^48^ Subgroup analysis found that ABC-type DLBCL patients who aged over 60 years with an IPI score of 3-5 points achieved the greatest benefit.^48^ A phase Ib/II trial indicated that DEL patients had a superior survival rate in the Pola combined with bendamustine and rituximab group compared with the bendamustine and rituximab group (mPFS 7.03 m vs 1.40 m, HR = 0.37).^49^ Loncastuximab tesirine is another ADC approved for R/R DLBCL, with a humanized anti-CD19 monoclonal antibody conjugated to a pyrrolobenzodiazepine dimer toxin. A phase 2 trial (LOTIS-2) on the safety and efficacy of Loncastuximab tesirine for the treatment of R/R DLBCL patients indicated that 70 out of 145 patients achieved a complete or a partial response, including 20 DEL patients.^50^ These encouraging results demonstrated that ADCs may be an alternative treatment for DEL.

## Chimeric antigen receptor therapy

Chimeric antigen receptor T-cell therapy (CAR-T) has been approved for the treatment of DLBCL after failure of ≥2 lines of therapy. A phase III trial (ZUMA-7) was conducted to evaluate the efficacy of Axicabtagene ciloleucel (axi-cel) and standard care (chemoimmunotherapy followed by autologous stem-cell transplantation) in the second-line treatment of early R/R DLBCL patients. The axi-cel group achieved a significantly longer PFS (14.7 vs 3.7 months) and median OS (not reached vs 31.1 months) compared with the standard care group, and this longer OS has also been observed in the DEL subgroup.^51^ A multicenter retrospective study evaluated the efficacy and prognosis of CAR-T therapy in patients with relapsed/refractory aggressive B-cell non-Hodgkin lymphoma. The study demonstrated that the overall response rate (ORR) was comparable among double-hit lymphoma, DEL, and other patient groups (ORR: 69% vs 64% vs. 66%, *P* = 0.8; complete response rate: 49% vs 42% vs 48%, *P* = 0.6). Similarly, median PFS (7.5 vs 6.2 vs 9.0 months, *P* = 0.35) and median OS (NR vs 19.1 vs 25.7 months, *P* = 0.8) were not significantly different. These findings suggest that CAR-T therapy may mitigate the adverse prognostic impact of MYC/BCL2 double expression in relapsed patients.^52^ Another retrospective study included 24 patients with B-cell lymphoma, 11 of whom were DEL patients, who received CD19/CD22 dual-targeted CAR-T therapy. The results showed that 14 patients achieved CR and 7 achieved partial remission 1 month after infusion. Moreover, survival analysis revealed that the double expression of MYC and BCL2 independently predicted PFS and OS.^53^

## Bispecific antibodies

The bispecific antibodies are a highly effective therapy that has been approved for at least 3 prior lines of therapy for DLBCL. Prior research demonstrated that bsAbs can also serve as a supplementary and salvage therapy following CAR-T or ASCT.^54^ A phase I/II study was conducted to evaluate the efficacy and safety of mosunetuzumab in previously untreated elderly DLBCL patients, and it was found that 43% of patients achieved CR, with an objective response rate (ORR) of 56%. Notably, patients with DEL had a greater ORR compared with those with double-hit/triple-hit lymphoma (43% vs 20%).^55^ A phase II clinical trial evaluated the efficacy of pretreatment with obinutuzumab followed by fixed-duration glofitamab for patients with DLBCL who had a median of 3 prior lines of therapy. The results revealed that 39% of patients achieved CR, and the ORR was 52%. Patients with the GCB subtype and DEL achieved the greatest benefit.^56^ A phase II study also found that DEL patients may benefit from the consolidation therapy with blinatumomab following standard R-CHOP.^57^ The abovementioned studies suggested that DEL patients may benefit from BsAbs.

## Autologous hematopoietic stem cell transplantation

Autologous hematopoietic stem cell transplantation (ASCT) can improve the survival rate in patients experiencing their first relapse of DLBCL.^58^ However, patients with R/R DLBCL undergoing ASCT experienced excessive death after at least 5-year following-up.^59^ Therefore, experts recommend that ASCT should be cautiously considered for patients who relapse more than 1 year after initial treatment or after CAR-T therapy.^60^ Whether DEL patients can benefit from ASCT remains controversial.

A retrospective study investigated whether DEL could benefit from consolidation therapy following standard first-line immunochemotherapy and found no significant difference in 5-year PFS (76.1% vs 81.8%) and OS (77.8% vs 80.3%) between the DEL group and the non-DEL group.^61^ This result was further confirmed by Taha et al.^62^ However, another multicenter retrospective study indicated that R/R DEL patients had inferior outcomes after standard ASCT.^63^ Furthermore, results of one rigorous retrospective study also indicated that DEL group had inferior 4-year PFS (48% vs 59%) and OS (56% vs 67%) compared with non-DEL group.^64^ It remains unclear whether DEL patients have a different prognosis compared with non-DEL patients following treatment with ASCT, as the available data are inconclusive.

## How to deploy troops and formations

DEL demonstrates biological and molecular heterogeneity, with prognostic variations depending on the lymphoma’s molecular context.^65^ Consequently, treatment approaches may differ across distinct patient populations. Molecular subtyping of DEL offers insights into personalized treatment strategies. A study showed that the MCD subtype had the highest proportion of DEL among all genetic subtypes. The study also revealed that DEL is primarily composed of the MCD, EZB, BN2, and other genetic subtypes.^66^ George et al. identified that the activation of the BCR-NF-κB signaling pathway is critical in the MCD and BN2 subtypes, which may benefit from BTK inhibitors. Moreover, BCL2 was essential in MCD, BN2, and EZB models, while BCL-XL was additionally critical in MCD, suggesting that agents such as venetoclax could be beneficial. The N1 subtype, characterized by gain-of-function NOTCH1 mutations, responded effectively to lenalidomide in DLBCL with inflammatory gene alterations, including the NOTCH pathway. Mutations in histone acetyltransferases CREBBP/EP300 are enriched in the EZB subtype, and with the upregulation of the histone deacetylation pathway, HDAC inhibitors may be effective in this subtype.^67-70^

Additionally, analysis of biopsies from the Phoenix trial revealed that patients aged ≤60 years with MCD and N1 subtypes experienced significant survival benefits. The 3-year EFS for younger patients (≤60 years) treated with ibrutinib plus R-CHOP was 100% in the MCD and N1 subtypes, whereas survival was markedly lower in patients receiving R-CHOP alone (42.9% and 50%, respectively).^71^ Zhao et al. conducted a phase II trial to evaluate the efficacy of genetic subtype-guided targeted agents (ibrutinib for MCD-like and BN2-like, lenalidomide for N1-like and NOS, decitabine for TP53 mutations, and tucidinostat for EZB-like) combined with R-CHOP (R-CHOP-X) in newly diagnosed intermediate- or high-risk DLBCL patients. The results showed that patients receiving R-CHOP-X achieved a superior CR rate (88% vs 66%) and improved survival outcomes (2-year PFS: 88% vs 63%; 2-year OS: 94% vs 77%) compared with the R-CHOP group.^72^ In conclusion, DEL demonstrates biological and molecular heterogeneity, with variations observed among patients. Genetic subtyping may guide the selection of treatment strategies; however, further rigorous and robust studies are needed to identify appropriate patient populations and optimize treatment approaches.

**Figure 1.**
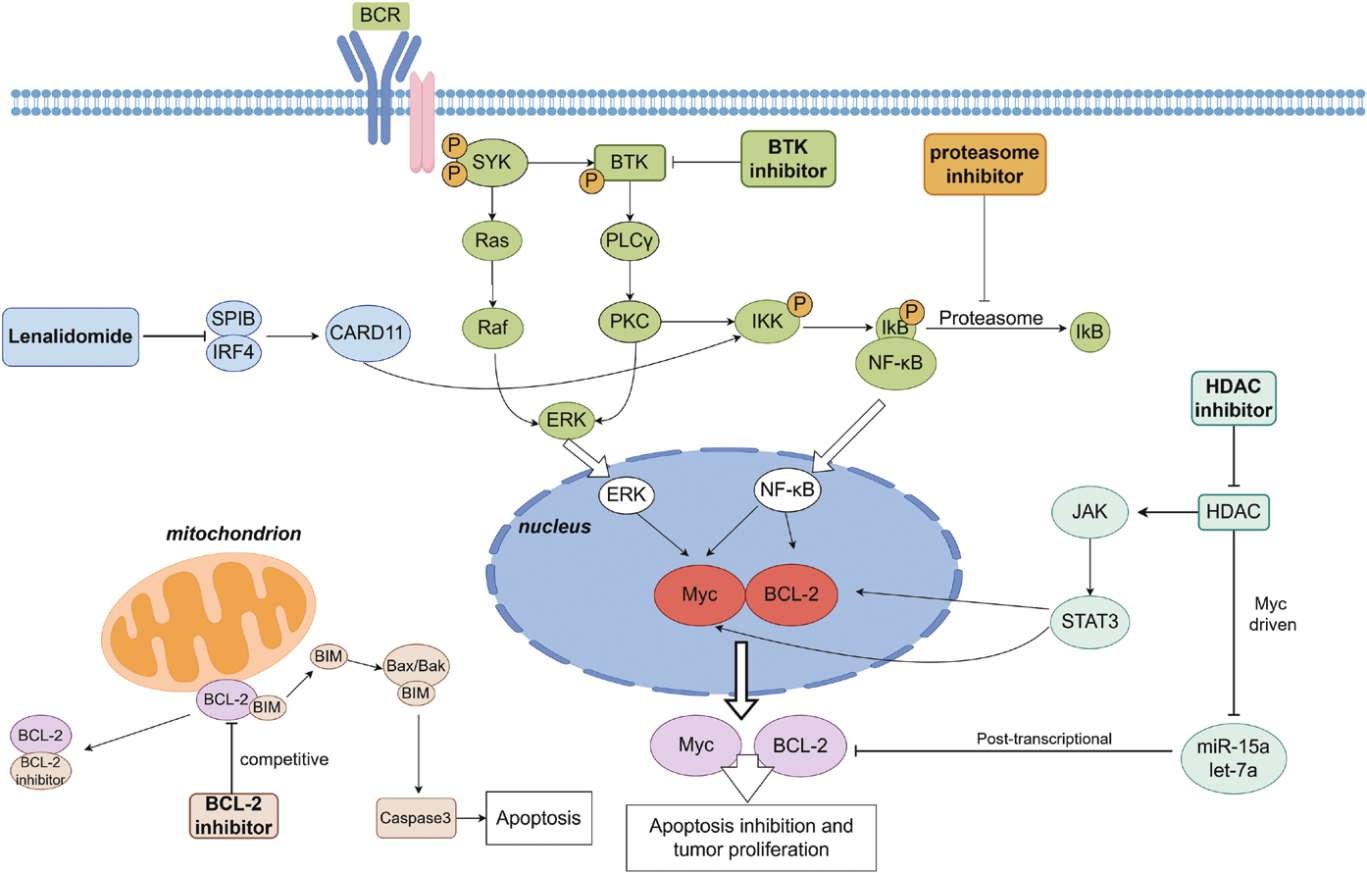
The molecular mechanism of potential small molecular drugs in DEL.

We have proposed potential drugs and treatment strategies targeting DEL in the abovementioned discussion (Table 1). The potential molecular mechanisms of some small molecular drugs are illustrated in Figure 1. However, effectively selecting and combining these treatments in clinical practice continues to be a challenge for clinicians. Some drugs demonstrate synergistic effects when used together or in combination with immunochemotherapy, which may provide a reference basis for the development of treatment plans. For instance, HDACi and BTKi could increase the efficacy of rituximab by upregulating CD20 surface expression and enhancing NK cell-mediated antibody-dependent cell-mediated cytotoxicity effect.^70,73^ The combination of Bcl-2 inhibitor with rituximab or CHOP can synergistically enhance their anti-tumor effect.^35,74^ Furthermore, HDACi could act synergistically with Bcl-2 inhibitor and BTKi to improve anti-tumor effects.^36,75^ Effectively improving the synergistic effects of these medications can result in a treatment plan that is greater than the sum of its parts.

**Table 1.** Clinical research on DEL.

Target or agent	Authors and Year	Clinical trial	Line of treatment	Combination therapy	Control Group	Clinical outcome	DEL/Total patients	Main 3/4 grade AEs
**BTKi**								
Zanubrutinib	Qiang He et al. 2022^22^	Phase II clinical trial (NCT05189197)	First-line	R-CHOP	NA	ORR (85.7%)	28/28	Hematological toxicities 42% Pneumonia 10%
Orelabrutinib	Yang Yang et al. 2022^23^	Phase II clinical trial (NCT05933967)	First-line	R-CHOP	NA	ORR (100%)	8/8	Neutropenia 62.5% Anemia34% Thrombocytopenia 25%
Ibrutinib	Johnson PWM et al. 2023^24^	Phase III clinical trial Phoenix study (NCT01855750)	First-line	R-CHOP	Placebo + R-CHOP	Improved EFS and OS in patients < 60 years of age	123/386	Febrile neutropenia 18.8% Pneumonia 6.7%
**HDACi**								
Tucidinostat	Mu-Chen Zhang et al. 2020^32^	Phase II clinical trial (NCT02753647)	First-line	R-CHOP	NA	ORR (94%), 2-year PFS (68%), 2-year OS (83%)	12/49	Neutropenia 84% Anemia 18% Infection 16%
	Weili Zhao et al. 2024^33^	Phase III clinical trial DEB study (NCT04231448)	First-line	R-CHOP	Placebo + R-CHOP	CR rate (73% vs. 61.8%), 2-year EFS rate (58.9% vs. 46.2%)	211/211	Pneumonia 15.6% 33 Hypokalemia 14.7% Neutropenia 60.2% Anemia19% Thrombocytopenia25.1%
**BCL-2i**								
Venetoclax	Matthew S. Davids et al. 2017^37^	Phase I clinical trial (NCT01328626)	Second-line	NA	NA	ORR (33.33%)	6/108	Anemia 15% Neutropenia 11% Thrombocytopenia 9%
Venetoclax	Andrew D Zelenetz et al. 2019^38^	Phase I clinical trial CAVALLI study (NCT02055820)	First-line	R/G-CHOP	NA	ORR (87.5%)	8/56	Neutropenia febrile neutropenia Thrombocytopenia Anemia 54.2%,33.3%,16.7%, and 12.5% in R-CHOP group 59.4%, 25.0%, 37.5%, and 31.3% in G-CHOP group
Venetoclax	Franck Morschhauser et al. 2021^39^	Phase II clinical trial CAVALLI study (NCT02055820)	First-line	R/G-CHOP	NA	CR rate (66%) 2-year PFS (72%) 2-year OS (90%)	80/206	Neutropenia 68% Febrile neutropenia 31% Thrombocytopenia 22% Anemia 24% Infections 23%
**PI**								
	Andrew J Davies et al. 2023^41^	Phase III clinical trial REMoDL-B study (NCT01324596)	First-line	R-CHOP	Placebo + R-CHOP	improved PFS	104/394	Neutropenia 29.3% Febrile neutropenia 12.8% Neuropathy 3.8%
**Immunomodulatory agents**								
Lenalidomide	Jason Westin et al. 2023^47^	Phase II clinical trial SMART trial (NCT02636322)	First-line	R + Ibrutinib 2 cycles + chemotherapy 6 cycles	NA	CR rate (95%), 2-year PFS (91%), 2-year OS (97%)	24/60	Neutropenia 53% Anemia 38% Febrile neutropenia 37% Thrombocytopenia 47% Rash 15% Diarrhea 13%
**ADCS**								
Polatuzumab vedotin	Hervé Tilly et al. 2022^48^	Phase III clinical trial POLARIX study (NCT03274492)	First-line	R-CHP	R-CHOP	2-year PFS (75.5% vs 63.1%)	139/440	Neutropenia 28.3% Anemia 12% Febrile neutropenia 13.8%
Polatuzumab vedotin	Laurie H Sehn et al. 2020^49^	Phase Ib/II clinical trial (NCT02257567)	Second-line	R/G + Bendamustine	R/G + Bendamustine	Median PFS (7.03 m vs 1.40 m)	11/39	Neutropenia 46.2% Anemia 28.2% Thrombocytopenia 41% Infections 23%
Loncastuximab tesirine	Paolo F Caimi et al. 2021^50^	Phase II clinical trial LOTIS-2 study (NCT03589469)	Second-line	NA	NA	ORR (48.3%)	20/145	Neutropenia 26% Anemia 10% Thrombocytopenia 18%
**CAR-T**								
Axicabtagene ciloleucel	Jason R Westin et al. 2023^51^	Phase III clinical trial ZUMA-7 study (NCT03391466)	Second-line	NA	Platinum-based chemotherapy	4-year OS rate (53.2% vs 45.6%, HR = 0.73)	57/180	Infections 16.5% Neutropenia 69% Anemia 30% Thrombocytopenia 15% Hypophosphatemia 18% Neurologic event 21%
**BsAb**								
Mosunetuzumab	Adam J. Olszewski et al. 2022^55^	Phase I/II clinical trial (NCT03677154)	First-line	NA	NA	ORR (43%)	12/54	Neutropenia 13%
Glofitamab	Michael J Dickinson et al. 2023^56^	Phase II clinical trial (NCT03075696)	Third-line	NA	NA	CR rate (39%)	15/154	Neutropenia 27%
Blinatumomab	Deborah A Katz et al. 2023^57^	Pase II clinical trial (NCT03023878)	First-line	R-chemotherapy	NA	ORR (89.3%)	10/28	Infections 10.7% Neutropenia 14.3%

Abbreviations: ASH: American Society of Hematology; AE: Adverse event; ORR: Objective remission rate; EFS: Event-free survival; OS: Overall survival; PFS, progressive-free survival; R, Rituximab; G, Obinutuzumab.

**Figure 2.**
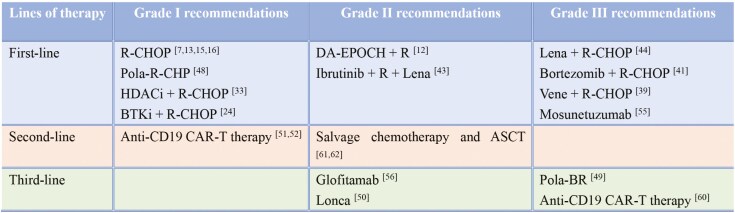
The preferred treatment recommendations for DEL according to the results of clinical trials and NCCN guidelines.

The National Comprehensive Cancer Network (NCCN) guidelines provided treatment recommendations based on the staging of DLBCL,^76^ whereas the Chinese Society of Clinical Oncology guidelines stratified treatment on the basis of age and IPI score.^77^ The treatment recommendations in these guidelines are based on the results of large clinical trials and are widely accepted and applied by clinicians. To date, the treatment consensus for DEL has not yet been established. The results of DEL clinical trials were summarized, and treatment recommendations for DEL were developed based on the phases and outcomes of these trials (Figure 2). A grade I recommendation represents a high level of evidence (large randomized controlled trials or rigorous meta-analysis). A grade II recommendation represents a slightly lower level of evidence (small randomized controlled trials, moderate-quality meta-analysis, or well-designed large retrospective studies). A grade III recommendation represents a lower level of evidence (uncontrolled single-arm clinical trials, expert opinions, or case reports).

For previously untreated DEL patients, chemoimmunotherapy remains the cornerstone treatment. The standard R-CHOP regimen or Pola-R-CHP regimen is preferentially recommended. Furthermore, treatment regimens, such as R-CHOP + X (where X includes HDACi, BTKi, Bcl-2 inhibitors, ICIs, and proteasome inhibitors) have exhibited efficacy and could serve as alternative options. CAR-T therapy is recommended as the preferred second-line therapy for R/R DEL, while ASCT may be appropriate for selected patients. Subsequent-line treatments may include BsAbs, ADC, salvage chemotherapy, etc. Nevertheless, determining the optimal treatment strategy warrants further investigation.

## Conclusion

In conclusion, DEL is a subtype of DLBCL with an unfavorable prognosis. Treating patients with multiple high-risk factors, especially the elderly, poses a significant challenge. Standard DLBCL treatments mainly provide limited benefits for these cases, and intensive chemotherapy frequently results in treatment interruptions. This manuscript outlines potentially effective drugs and treatment regimens identified in clinical trials for DEL and formulates treatment recommendations based on the available evidence levels. We believe that this study may provide valuable insights into the precise treatment of DEL.

## Data Availability

The data supporting the conclusions of this manuscript will be made available by the authors.
